# Supplementary material for: Fabrication and Mechanical Performance of Non-Crimp Unidirectional Jute-Yarn Preform-Based Composites
Source: Molecules. 2021 Nov 3;26(21):6664. doi: 10.3390/molecules26216664 (PMC8587098; doi:10.3390/molecules26216664)
Supplement: Supplementary file 1 [file molecules-26-06664-s001.zip › molecules-1439462-supplementary.pdf]

# Supporting Information

## Fabrication and mechanical performance of non-crimped unidirectional jute yarn preform based composites

**Yeasin Ali,[1] Atik Faisal,[2] Abu Saifullah,[3] Hom N Dhakal,[3] Shah Alimuzzaman[4] and Forkan Sarker[1*]**

[1] Department of Textile Engineering, Dhaka University of Engineering &Technology, DUET, Gazipur-1700, Bangladesh
[2] Department of Yarn Engineering, Bangladesh University of Textiles, Dhaka-1208, Bangladesh
[3] Advanced Polymers and Composites (APC) Research Group, School of Mechanical and Design Engineering, University of Portsmouth, Portsmouth PO1 3DJ, UK
[4] Department of Fabric Engineering, Bangladesh University of Textiles, Dhaka-1208, Bangladesh

* Correspondence: Forkan Sarker, UN Fellow, Commonwealth Scholar, Associate professor, Department of Textile Engineering, Dhaka University of Engineering and Technology, Email: forkan@duet.ac.bd, Cell No: +8801312717271

### Supporting Information 1

Different linear density of yarns has been used in this study. In these three types of yarn based on their linear density ( 20,25 and 30 lbs/spyndle ) were used. Digital images of the three yarn packages from left to right can be seen in Figure S1.

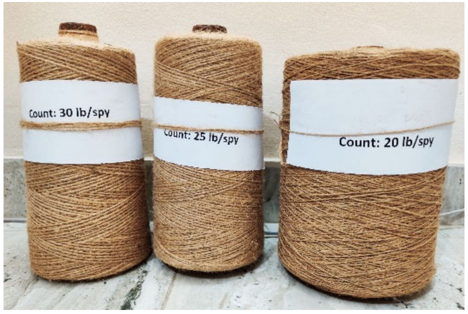


**Figure S1.** Digital images of jute yarns used in this study where the yarn liner densities from left are 30, 25 and 20 pound/spyndle.

**Supporting Information 2**

Tensile and flexural properties novel UD jute yarn composites are reported in Table S1. A significant different in the tensile properties is observed in this study among three different qualities of yarn-based jute/polyester composites. Stiffness of the UD jute composites has almost 4 times higher than the traditional woven composites. Similar trend is also observed in the bending properties. In this study we found that yarn with 25 lbs/spyndle offer highest mechanical properties than the other composites made from 20 and 30 lb/spyndle (see Table S1).

**Table S1.** Tensile and flexural properties of natural jute yarn composites made from UD and woven architecture.

| Composite type | Tensile Strength (MPa) | Tensile Modulus (GPa) | Flexural Strength (MPa) | Flexural Modulus (GPa) |
|---|---|---|---|---|
| W20 | 54 (±5) | 2.5 (±0.2) | 70 (±7) | 2.7 (±0.25) |
| UD20 | 93.57 (±11) | 5.66 (±0.74) | 172.18 (±9) | 5.06 (±0.3) |
| UD25 | 132.79(±12) | 8.31 (±1) | 171.56 (±13 | 6.44 (±0.13) |
| UD30 | 125.60(±7) | 7.80 (±0.57) | 178.34 (±6) | 5.83 (±0.24) |
